# Combined Chemotherapy and Immunotherapy Induction for Screening of Patients with Cervical Esophageal Carcinoma for Subsequent Local Treatment: A New Treatment Paradigm

**DOI:** 10.1245/s10434-024-15843-3

**Published:** 2024-07-26

**Authors:** Liang Dai, Ya-Ya Wu, Yan Sun, Rong Yu, Wan-Pu Yan, Yong-Bo Yang, Hong Cheng, Yi-Mei Gao, Bin Zhang, Ke-Neng Chen

**Affiliations:** 1 Key Laboratory of Carcinogenesis and Translational Research (Ministry of Education), The First Department of Thoracic Surgery, Peking University Cancer Hospital and Institute, Peking University School of Oncology, Beijing, China; 2 Department of Radiation Oncology, Peking University Cancer Hospital and Institute, Beijing, China; 3 Key Laboratory of Carcinogenesis and Translational Research (Ministry of Education), Department of Head and Neck Surgery, Peking University Cancer Hospital and Institute, Beijing, China

**Keywords:** Cervical esophageal carcinoma, Combined chemotherapy and immunotherapy, Local treatment, Overall survival, Induction therapy

## Abstract

**Background:**

Definitive chemoradiotherapy is recommended as the primary treatment for cervical esophageal carcinoma (CEC). However, local control rates remain unsatisfactory for some patients. Therefore, in this study, we introduced a new treatment paradigm for individuals with CEC, customizing the choice between subsequent local treatments based on their response to induction chemotherapy and immunotherapy.

**Patients and Methods:**

Induction treatment comprised two to four cycles of chemotherapy combined with programmed cell death protein 1 (PD-1) inhibitors. Patients achieving complete response (CR) or near CR after induction treatment underwent definitive chemoradiotherapy (dCRT), while those not achieving CR or near CR underwent surgical resection.

**Results:**

Among the 40 eligible patients, 14 (35.0%) achieved a CR or near CR after induction treatment. Of the ten patients achieving a CR or near CR, one developed an esophageal fistula after dCRT (10.0%). Among the eight non-CR or non-near CR patients receiving chemoradiotherapy, six developed esophageal fistula (75.0%). Among the 26 patients who did not achieve CR or near CR after induction treatment, the 1-year cancer specific survival (CSS) rates were 93.3% [95% confidence interval (CI) 0.815–1%] for the 18 patients in the surgery group, and 71.4% (95% CI 0.447–1%) for the 8 patients in the chemoradiotherapy group (*p* = 0.027). The overall laryngeal preservation rate was 85.0% (34/40), with a functional laryngeal preservation rate of 77.5% (31/40).

**Conclusion:**

The approach consisting of combined immunotherapy and chemotherapy successfully identified patients who were responding well to induction treatment and who were sensitive to radiotherapy, for chemoradiotherapy; thus, improving laryngeal preservation rates. In addition, it also identified patients with poor responses to induction treatment and radiotherapy, for timely surgery; hence, reducing radiotherapy complications and enhancing survival.

Cervical esophageal cancer (CEC) is relatively rare, accounting for approximately 5% of all cases of esophageal cancer.^1^ It is primarily characterized by squamous cell carcinoma.^2^ Typically diagnosed at an advanced local stage, CEC often involves crucial surrounding structures and is associated with lymph node metastasis. Given the significant surgical challenges associated with CEC, some patients may require laryngectomy, resulting in the loss of speech. Furthermore, the absence of long-term survival data specifically tied to surgical interventions has led the National Comprehensive Cancer Network (NCCN) guidelines to recommend definitive chemoradiotherapy (dCRT) as the initial treatment for CEC.^3^

However, post-dCRT, a substantial number of patients with CEC experience high rates of local control failure, and long-term survival outcomes are frequently unsatisfactory.^4,5^ For those facing treatment failure, the options are limited to high-risk salvage surgery or losing the opportunity for surgical resection. With advances in surgical technology, definitive surgical procedures offer a partial remedy for these challenges. Consequently, the current ideal approach is to identify patients that are sensitive to chemoradiotherapy for dCRT and those that are not, for planned surgical interventions. Nakata et al.^6^ introduced a “chemotherapy selection” scheme whereby induction chemotherapy is followed by dCRT for tumors with regression exceeding 30%, and surgery for those with less than 30% regression. The results revealed a higher 2-year survival rate in the chemotherapy selection group compared with the initial dCRT group (65.1% versus 40.0%). Similarly, Urba, S. et al.^7^ proposed dCRT for tumors with regression exceeding 50% after induction treatment, and surgery for those with less than 50% regression. The 2021 American Society of Clinical Oncology (ASCO)-reported CROS study^8^ also achieved favorable outcomes by selecting different local treatments based on responses to induction therapy.

There is currently insufficient evidence to demonstrate prolonged overall survival (OS) among patients with esophageal cancer receiving chemoimmunotherapy. However, the short-term antitumor effects [pathological complete response (pCR) rates] observed in the literature surpass those of induction chemotherapy alone.^9–13^ Since 2020, our center has employed chemotherapy combined with immunotherapy as an induction screening treatment for the subsequent local treatment of CEC. In this study, we compared complications, laryngeal preservation rates, and survival outcomes among patients that underwent different local treatments after induction therapy, aiming to identify suitable candidates for chemoradiotherapy or surgery.

## Patients and Methods

### Patients

The study recruited patients with CEC consecutively treated at the Department of Thoracic Surgery, Peking University Cancer Hospital, from May 2020 to December 2022.

The inclusion criteria included (1) initial pathological diagnosis of squamous cell carcinoma, (2) tumor located in the cervical esophagus, (3) initial clinical staging as cT1N+M0/1 or cT2-4aN+/−M0/1 (distant metastasis limited to supraclavicular lymph nodes), and (4) treatment involving induction chemotherapy and immunotherapy.

**Fig. 1:**
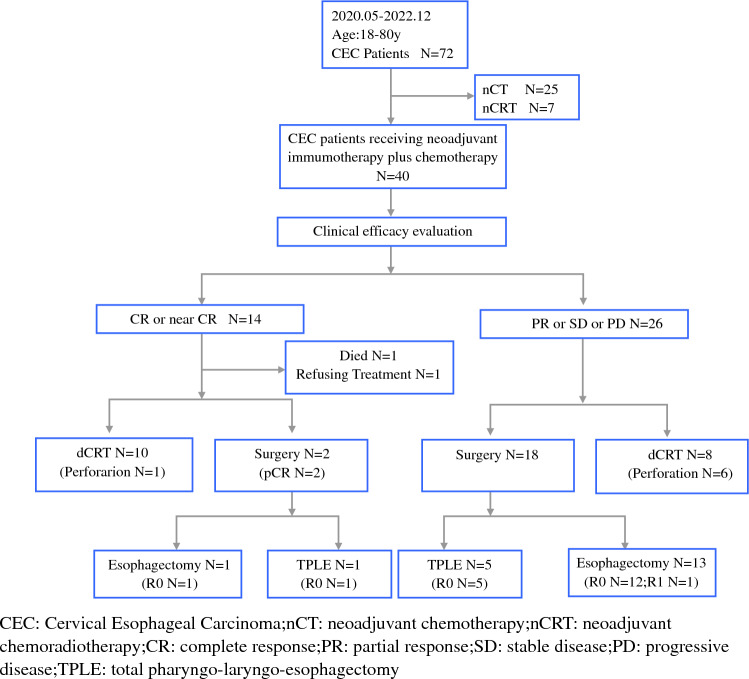
Patient enrollment and treatment workflow

The exclusion criteria included induction treatment involving chemoradiotherapy or chemotherapy alone. Detailed criteria can be found in Fig. 1. The study received approval from the Ethics Committee of Peking University Cancer Hospital (ethics number 2023YJZ70).

CEC was defined as a tumor with the upper margin within 3 cm of the esophageal inlet during endoscopy, or visible above the sternal notch on contrast radiography. It could or could not involve the lower pharynx and might coexist with thoracic esophageal cancer.

### Induction Treatment and Efficacy Evaluation

Induction treatment involved chemotherapy combined with programmed cell death protein 1 (PD-1) inhibitors. The chemotherapy regimen comprised cisplatin (75mg/m^2^) or carboplatin [area under the curve (AUC) of 5], combined with paclitaxel (175mg/m^2^), administered every 21 days for one cycle. After two cycles, an enhanced computed tomography (CT) of the neck and chest, enhanced esophageal magnetic resonance imaging (MRI), and, if necessary, positron emission tomography (PET)–CT were performed to assess the tumor’s objective response (using RESIST1.1^14^). Patients with inadequate response after two induction cycles underwent prompt surgical intervention. In contrast, for those that exhibited substantial tumor regression, induction treatment combining chemotherapy and immunotherapy was extended to three to four cycles.

Patients with significant tumor regression on imaging and who were considered to have achieved complete response (CR) or near-complete response (near CR) underwent esophagogastroduodenoscopy, and tissue samples were biopsied. CR or near CR was defined as follows: no definite mass on imaging, PET–CT showing no or only mild metabolic activity in the primary lesion, endoscopy revealing no distinct mass but mucosal repair or scar-like changes, and biopsy showing either no malignant cells (CR) or the presence of minimal malignant tumor cells (near CR) (Appendix 1).

### Local Treatment

Patients with CR or near CR after induction therapy received radical chemoradiotherapy. Surgery is recommended for patients who do not achieve CR or near CR. However, the final local treatment strategy (surgery or chemoradiotherapy) after induction therapy is determined jointly by the doctor and the patient.

### Surgery

Following induction treatment, a multidisciplinary assessment was performed to determine its efficacy. For patients not achieving CR or near CR, surgery was conducted within 4–10 weeks of the last cycle of induction treatment. The surgical approach encompassed a three-incision esophagectomy, with or without pharyngolaryngectomy. Preservation of the larynx was considered when the primary tumor did not invade the trachea, the upper margin was 2 cm or more from the esophageal inlet, and when the surgeon deemed laryngeal preservation feasible. We used preoperative endoscopic clips to locate the upper edge of the tumor and improved the anastomosis method (transverse section of the cervical trachea during surgery to help expose the esophageal entrance, followed by tracheal anastomosis and prophylactic tracheostomy). Technically, we were able to achieve anastomosis within 2 cm of the esophageal entrance while preserving the larynx. However, tumors often have longitudinal invasion under the mucosa. Based on our experience, a distance of 2 cm from the upper edge of the tumor to the esophageal entrance is a technically accessible and reliable standard for laryngeal preservation surgery in oncology.

The stomach served as the substitute organ. Those opting for laryngeal preservation underwent preoperative endoscopy to locate and clip the tumor’s upper margin, with esophagography to confirm the position.

### Concurrent Chemoradiotherapy

Patients achieving CR or near CR then underwent dCRT. The chemotherapy regimen involved carboplatin (AUC = 2) combined with paclitaxel (50 mg/m^2^), administered from the first day of radiotherapy, with weekly doses for a total of six administrations. Radiotherapy was delivered once daily, five times weekly, and entailed 2 Gy per fraction up to a cumulative dose of 60 Gy in 30 fractions. Three-dimensional conformal external beam radiation therapy was uniformly applied. The gross tumor volume (GTV) was delineated on the basis of pretreatment endoscopy, esophagography, CT, MRI, PET–CT, and other imaging studies to include the primary esophageal lesion and metastatic lymph nodes. The clinical tumor volume (CTV) included a margin of 3 cm in the superior–inferior direction and 0.5–1 cm in the anterior–posterior and left–right directions from the GTV. The planning tumor volume (PTV) was a three-dimensional isotropic expansion of 5 mm from the CTV.

### Outcome Points

The outcome points included overall survival (OS), cancer specific survival (CSS), laryngeal preservation (LP) rate, laryngeal function preservation (LFP) rate, and LFP survival. The OS was defined as the time from diagnosis to either the patient’s death or their last follow-up. CSS was defined as the time from diagnosis to either tumor-specific death or their last follow-up. LP rate was the percentage of patients who did not undergo laryngectomy out of the total patients. LFP rate was the percentage of patients with maintained laryngeal function out of the total patients. Endpoint events included language function loss, permanent tracheostomy, and laryngectomy. LFP survival was the time from diagnosis to the loss of laryngeal function. Endpoint events comprised death, speech loss, permanent tracheostomy, and laryngectomy. Adverse reactions were assessed in accordance with the Common Terminology Criteria for Adverse Events (CTCAE) version 5.0 from the National Cancer Institute [15].

### Data Analysis

R language was utilized for all data analyses. Categorical variables were presented as frequencies and percentages, while continuous variables were articulated as mean ± standard deviation. Survival analysis was done using Kaplan–Meier plots, and the log-rank test discerned differences among the clinical features of different groups. Statistical significance was set at *p* < 0.05.

## Results

### Patients

Between May 2020 and December 2022, a total of 72 cases of CEC were treated at our hospital. Based on the inclusion criteria (Fig. 1), 40 patients were ultimately enrolled in the study. Among these, 31 patients (77.5%) were male, while 9 patients (22.5%) were female. The average age of the entire cohort was 64.68 ± 8.87 years. The clinical stages were III and IV (87.5%), with 7 cases demonstrating supraclavicular lymph node metastasis (17.5%). Preoperatively, 15 cases (37.5%) received 2 cycles of chemotherapy combined with immunotherapy, 12 cases (30.0%) received 3 cycles, and 13 cases (32.0%) received 4 cycles.

Among the 40 patients in this study, 37 had PD ligand 1 (PD-L1) expression measurement, including 18 patients with a combined positive score (CPS) < 10 and 19 patients with CPS ≥ 10. Among the 18 patients with CPS < 10, 7 patients were reevaluated with progressive disease (PD) and stable disease (SD), 6 with partial response (PR), and 5 with CR after chemotherapy combined with immunotherapy. Among the 19 patients with CPS ≥ 10, 7 patients were reevaluated with SD, 6 with PR, and 6 with CR (*p* = 0.969) (Table 1).

**Table 1:** General clinical and pathological information

Clinicopathologic factors	No. of patients
	*N* (%)
*Gender*	
Man	31 (77.5%)
Female	9 (22.5%)
Age (mean ± SD)	64.68 ± 8.87
*ECOG*	
0	35 (87.5%)
1	4 (10.0%)
2	1 (2.5%)
*Differentiation grade*	
Poor	19 (47.5%)
Moderate	19 (47.5%)
Well	2 (5.0%)
*Clinical stage*	
I	1 (2.5%)
II	4 (10.0%)
III	13 (32.5%)
IV	22 (55.0%)
*cT*	
1	1 (2.5%)
2	4 (10.0%)
3	19 (47.5%)
4	16 (40.0%)
*cN*	
0	3 (7.5%)
1	18 (45.0%)
2	18 (45.0%)
3	1 (2.5%)
*cM*	
0	33 (82.5%)
1	7 (17.5%)
*Cycles of neoadjuvant chemotherapy*	
2	15 (37.5%)
3	12 (30.0%)
4	13 (32.0%)

### Effectiveness and Safety of Induction Therapy

**Fig. 2:**
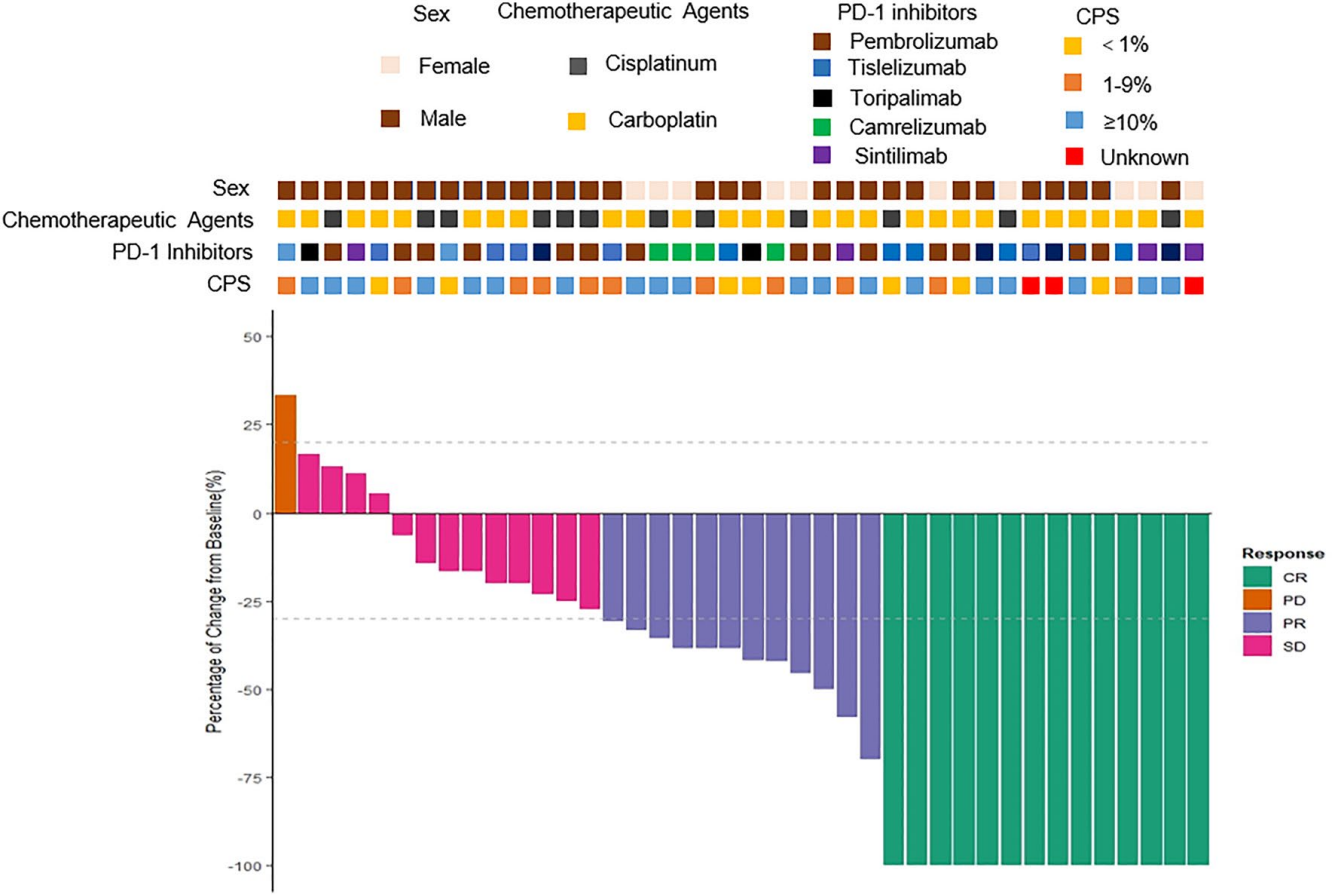
Waterfall chart

Following two to four cycles of chemotherapy combined with immunotherapy, 14 (35.0%) patients achieved a CR or near CR, and 12 (30.0%) achieved partial response (PR). Stable disease (SD) was observed in 13 patients (32.5%), while 1 patient (2.5%) experienced progressive disease (PD; Fig. 2).

Among the 40 patients, 16 (40.0%) developed treatment-related adverse reactions, with no unreported toxicities. The most common were gastrointestinal reactions (17.5%) and bone marrow suppression (17.5%). Seven cases (17.5%) experienced grade 3 or higher adverse reactions. Specifically, bone marrow suppression occurred in five cases (12.5%), gastrointestinal reactions in two cases (5.0%), and immune-mediated myocarditis in one case (2.5%). Apart from one case that resulted in acute right heart failure and death due to immune-mediated myocarditis, the remaining complications improved after appropriate symptomatic interventions (Table 2).

**Table 2:** Treatment related adverse events

Adverse events	Grade1–2	≥ Grade3
	*N* (%)	*N* (%)
Inductive treatment (*N* = 40)	9 (22.5)	7 (17.5)
Myelosuppression	2 (5.0)	5 (12.5)
Gatrointestinal reaction	5 (12.5)	2 (5.0)
Pneumonia	1 (2.5)	0 (0.0)
Liver function injury	2 (5.0)	0 (0.0)
Thyroid dysfunction	4 (10.0)	0 (0.0)
Rash	3 (7.5)	0 (0.0)
Myocarditis	0 (0.0)	1 (2.5)
Surgery(*N* = 20)	4 (20.0)	8 (40.0)
Gatrointestinal reaction	4 (20.0)	0 (0.0)
Pneumonia	1 (5.0)	4 (20.0)
Anastomotic Fistula	0 (0.0)	4 (20.0)
Recurrent nerve paralysis	0 (0.0)	2 (10.0)
Incision infection	2 (10.0)	0 (0.0)

### Local Treatment

Patients achieving a CR or near CR following neoadjuvant therapy were recommended for chemoradiotherapy after multidisciplinary team (MDT) discussions. Alternatively, those who did not attain a CR or near CR were advised to undergo surgical treatment. Among the 14 patients achieving a CR or near CR, one patient succumbed to immune-mediated myocarditis, and another patient declined further during intervention. A total of ten patients opted for dCRT, while two underwent surgery (one with concurrent laryngectomy), and all exhibited pCR upon postoperative pathological examination. Among the 26 patients without a CR or near CR, 18 underwent surgical treatment (including 5 with concurrent laryngectomy), and 8 received chemoradiotherapy (Fig. 1).

For the 18 patients receiving definitive radiotherapy postinduction treatment, the average dose to the primary lesion was 60.57 ± 6.27 Gy, and for involved lymph nodes, it was 59.73 ± 5.52 Gy. Of the 10 CR or near CR patients among the 18 patients, 7 (70.0%) experienced treatment-related adverse reactions, with reflux esophagitis being the most prevalent (60.0%). Two patients (20.0%) developed grade 3 or higher adverse reactions, including bone marrow suppression (10.0%) and esophageal perforation (10.0%). In the eight non-CR or non-near CR patients, 6 (75.0%) reported treatment-related adverse reactions, with 5 (62.5%) encountering grade 3 or higher toxicities, all related to esophageal perforation (Table3).

**Table 3:** Treatment-related adverse reactions during Concurrent Chemoradiotherapy

	Concurrent chemoradiotherapy	
	CR	Non-CR
	*N* = 10 (%)	*N* = 8 (%)
*Myelosuppression*		
Grade1–2	3 (30.0)	1 (12.5)
≥ Grade 3	1 (10.0)	0 (0.0)
Gatrointestinal reaction		
Grade1–2	2 (20.0)	2 (25.0)
≥ Grade 3	0 (0.0)	0 (0.0)
Pneumonia		
Grade1–2	1 (10.0)	2 (25.0)
≥ Grade 3	0 (0.0)	0 (0.0)
Esophagitis		
Grade1–2	5 (50)	1 (12.5)
Esophageal Perforation	1 (10)	5 (62.5)
Total		
Grade1–2	5 (50)	1 (12.5)
≥ Grade 3	2 (20)	5 (62.5)

A total of 20 patients underwent surgical resection post induction treatment, with 6 undergoing concurrent laryngectomy and 14 opting for laryngeal preservation procedures. The average distance of the tumor to the upper margin in patients undergoing laryngeal preservation procedures was 1.85 ± 1.23 cm. Among the 20 surgical patients, 12 (60.0%) experienced treatment-related adverse reactions, primarily pneumonia (25.0%). Eight patients (40.0%) experienced grade 3 or higher adverse reactions, including pneumonia in four cases (20.0%), anastomotic fistula in four cases (20.0%), and recurrent laryngeal nerve injury in two cases (10.0%). All patients showed improvement after symptomatic treatment, with no perioperative deaths recorded (Table 2).

### Survival

The median follow-up duration for the entire patient cohort was 15 months. The 1-year OS rate for all patients reached 77.9% [95% confidence interval (CI) 0.653–0.928%], with a median OS of 21 months (95% CI 0.233–0.875 months). In terms of CSS, the 1-year rate was 83.2% (95% CI 0.717–0.965%), and the median CSS was not reached (Appendix 2). After induction treatment, the 1-year OS rates for patients with CR or near CR and non-CR or non-near CR were 69.3% (95% CI 0.480–0.999%) and 83.5% (95% CI 0.699–0.999%), respectively (*p* = 0.53). The 1-year CSS rates were 77.9% (95% CI 0.587–1%) and 86.9% (95% CI 0.740–1%), respectively (*p* = 0.86) (Appendix 3).

For patients with CR or near CR undergoing dCRT and non-CR or non-near CR patients undergoing combined surgery postinduction treatment, the 1-year OS rates were 68.6% (95% CI 0.445–1%) and 93.3% (95% CI 0.815–1%), respectively (*p* = 0.079), while the 1-year CSS rates were 80.0% (95% CI 0.587–1%) and 93.3% (95% CI 0.851–1%), respectively (*p* = 0.65) (Appendix 4).

**Fig. 3:**
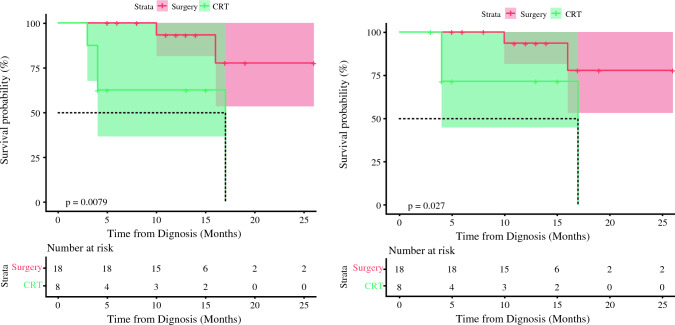
Comparison of OS and CSS between non-CR patients receiving chemoradiotherapy or surgery

In non-CR or non-near CR patients receiving surgery or chemoradiotherapy postinduction treatment, the 1-year OS rates were 93.3% (95% CI 0.815–1%) and 62.5% (95% CI 0.365–1%), respectively (*p* = 0.008). The 1-year CSS rates were 93.3% (95% CI 0.815–1%) and 71.4% (95% CI 0.447–1%), respectively (*p* = 0.027) (Fig. 3).

### LFP Rate

Among the 20 patients undergoing surgery, 6 also underwent concurrent laryngectomy. In the group of 18 patients receiving chemoradiotherapy, 7 experienced perforations, 3 of whom suffered voice loss. The overall laryngeal preservation rate for all patients was 85% (34/40), with an LFP rate of 77.5% (31/40). In contrast, in the 26 non-CR or non-near CR patients, 5 out of 18 surgical patients underwent concurrent laryngectomy, resulting in an LFP rate of 72.2% (13/18). Among the eight patients receiving dCRT, three experienced voice loss, resulting in an LFP rate of 62.5% (5/8).

**Fig. 4:**
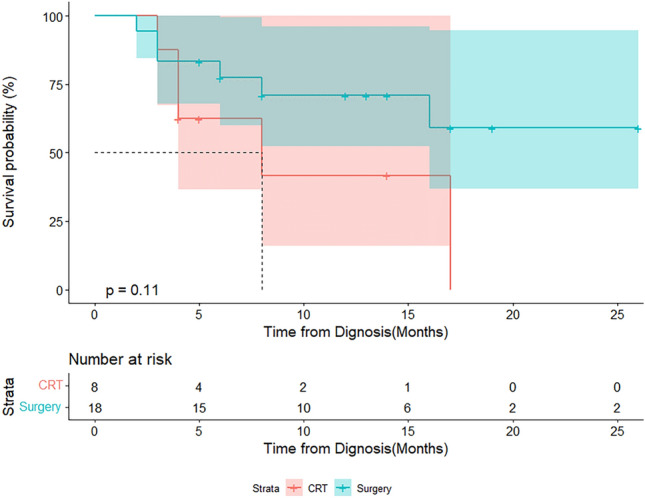
LFP survival rates between non-CR patients receiving chemoradiotherapy or surgery

The 1-year LFP survival rate for the entire cohort was 62.2% (95% CI 48.2–80.3%) (Appendix 5). When comparing the CR or near CR and non-CR or non-near CR patients, the 1-year LFP survival rates were 61.9% (95% CI 40.2–95.3%) and 62.7% (95% CI 45.9–85.8%), respectively (*p* = 0.80) (Appendix 6). For non-CR or non-near CR patients undergoing chemoradiotherapy or surgery, the 1-year LFP rates were 41.7% (95% CI 15.9–100.0%) and 70.9% (95% CI 52.3–96.2%), respectively (*p* = 0.11) (Fig. 4).

## Discussion

The findings of this study suggest that “combined induction chemotherapy and immunotherapy” effectively identifies patients with CEC who will respond well or poorly to subsequent chemoradiotherapy. In comparison with dCRT, surgery proved beneficial by reducing complications, enhancing local control, and improving overall survival in patients who are less responsive to chemoradiotherapy. Conversely, when compared with surgical intervention, dCRT increased laryngeal preservation rates and maintained the esophagus, for patients who were responsive. Notably, this study is the first to report on the use of chemotherapy combined with immunotherapy to guide subsequent local treatment strategies in CEC.

Although drawing definitive survival advantages from a small sample study is challenging, our results appear promising when compared with outcomes reported in earlier studies. The 1-year OS rate was 77.9%, and after excluding the three cases that succumbed to pneumonia during the coronavirus disease 2019 (COVID-19) pandemic, the CSS rate was 83.2%. Zhao, D. et al. reported a 1-year OS of 78.3% for 82 patients with CEC after dCRT.^5^ Meanwhile, Dai, K.Y. et al. reported a 1-year and 2-year OS of approximately 70% and 50.6%, respectively.^16^ Additionally, our current patient cohort, despite having later clinical stages, demonstrated promising survival outcomes compared with several other studies, where later stages often correlated with poorer survival.

An increasing body of evidence underscores substantial disparities in outcomes between responders and nonresponders, following induction therapy.^7,17^ This highlights the promising strategy of induction screening, to enhance the treatment of poorly responsive patients. To stratify patients using induction therapy, the selected treatment must be effective and well-tolerated, and immunotherapy meets these criteria, precisely. In our study, the rate of achieving a CR or near CR after combined chemotherapy and immunotherapy reached 35%, with an overall response rate (ORR) of 65%, resembling other neoadjuvant immunotherapy combined with chemotherapy approaches in esophageal cancer.^9–13^ In addition, the toxicity tolerance of combined chemotherapy and immunotherapy was good, and no new or unreported toxic side effects were observed. The most common grade 3 and above treatment-related adverse reactions were hematologic toxicities, mostly related to chemotherapy. This outcome aligns with the safety profile reported in other studies on chemotherapy combined with immunotherapy.^9–13^

For patients achieving a CR or near CR after induction therapy, we opted for dCRT. There is currently a lack of direct comparison data between dCRT and surgery for CEC. However, organ preservation treatment in patients with early-stage disease or significant clinical relief after induction therapy has shown favorable outcomes. The JCOG9708 study^18^ reported a 4-year OS rate of 80.5% and disease-free survival (DFS) rate of 68.1% for stage I esophageal squamous cell carcinoma. Building on this study, the JCOG0502 study^19^ indicated no significant differences in 5-year OS (86.5% versus 85.5%) and progression-free survival (PFS) (81.7% versus 71.6%) between patients receiving surgery and those receiving chemoradiotherapy. The CROS study^8^ explored the outcomes of neoadjuvant chemotherapy with the docetaxel, cisplatin, and 5–fluorouracil regimen followed by chemoradiotherapy in responders and surgery in non-responders. It showed a 3-year OS of 83.7% for the chemoradiotherapy group and 62.8% for the surgery group, with an overall organ preservation survival rate of 56.8% and 45.3% at 1 and 3 years for the entire cohort. A meta-analysis by van der Wilk et al.^20^ found no significant difference in long-term survival between organ preservation strategies and surgical resection in patients achieving complete clinical response (cCR) after neoadjuvant chemotherapy. Valmasoni et al.^21^ observed that additional surgery in patients achieving cCR after dCRT in CEC did not improve long-term survival. The results of the SANO study^22^ were announced at the European Society for Medical Oncology meeting in 2023: there was no statistical difference between active monitoring and standard surgery for patients with CCR, neither OS nor DFS.

We attribute the relatively favorable survival outcomes in this study to the successful early identification of patients who might fail subsequent dCRT through “combined chemotherapy and immunotherapy induction” as well as the timely follow-up with surgical intervention. For patients who did not achieve a CR after induction therapy, the satisfactory survival rate after surgery compared with the survival rate after chemoradiotherapy (1-year OS rate of 93.3% versus 62.5%) highlighted the beneficial role of timely surgical intervention. Among the seven patients who underwent CRT, one patient (1/10) had CR or near CR after induction therapy. During follow-up, it was confirmed by endoscopy that there was an esophagotracheal fistula, but no residual cancer cell was found in the biopsy specimen around the fistula. The perforation healed after conservative treatment with fasting and water deprivation for 1 month, confirming that the esophageal perforation may be caused by tumor regression after radiotherapy, rather than tumor progression. The remaining six cases with perforation were of patients (6/8) who did not obtain CR or near CR after induction therapy. They experienced perforation due to tumor progression during radiotherapy or within 6 months after radiotherapy, and the tumors were not controlled even after palliative treatment or second-line chemotherapy. The median OS was 4 months (OS range 3–17 months). Additionally, we can draw valuable insights from the failure patterns observed in the treatment of CEC with dCRT. Zhang et al.^23^ analyzed 102 cases of patients with CEC, and revealed that 31.4% experienced local treatment failure, and 25.5% experienced regional treatment failure. Similarly, Zhao et al.^5^ reported that 46.3% of the patients experienced local failure, and 28.0% experienced regional treatment failure after radical radiotherapy in 82 cases of CEC. These relatively elevated rates of local and regional treatment failure suggest deficiencies in the effectiveness of local treatment with dCRT for CEC. Radical surgical resection may, to some extent, address these shortcomings. In a prior study by Nakata et al.,^6^ 15 cases of local control failure were reported in the radiotherapy group for CEC, with 11 undergoing salvage surgery. The 5-year OS rate in the salvage surgery group was 64.8%, significantly surpassing the 44% OS rate observed in the conservative treatment group.

In the context of treating CEC, beyond long-term survival, the laryngeal preservation rate stands out as a crucial metric. Our study showcased an impressive laryngeal preservation rate of 85%, significantly surpassing reported rates for both dCRT and radical surgery.^24–27^ The evolution of laryngeal preservation has transitioned from merely safeguarding anatomical structures to emphasizing the retention of laryngeal function, that is, the survival rate without laryngoesophageal functional impairment (i.e., for patients without laryngectomy and tracheostomy). In our investigation, the 1-year LFP survival rate reached 62.2% (95% CI 48.2–80.3%). For patients undergoing dCRT for CEC, even if they retain the normal anatomical structure of the larynx, many may experience voice loss. The LFP survival rate in these cases may be lower than initially estimated.^28,29^ Notably, in our study, the 1-year LFP rate for patients who pursued dCRT after induction treatment without achieving complete response was only 41.7% (95% CI 15.9–100.0%). However, opting for surgery elevated this rate to 70.9% (95% CI 52.3–96.0%). Additionally, among the eight patients who underwent dCRT without achieving a CR, six experienced perforations, and the rate of grade 3 or higher complications was substantial at 75%. In contrast, patients who underwent surgery demonstrated a commendable R0 resection rate of 94.4%, accompanied by a 40% occurrence of grade 3 or higher complications. A retrospective analysis by Makino et al.^30^ involving 100 cases of patients with CEC revealed that laryngeal preservation surgery demonstrated comparable perioperative complication risks and long-term survival with surgery combined with laryngectomy. Our study results reinforce the notion that anatomical preservation does not equate to functional preservation. Therefore, whether considering long-term survival, laryngeal function preservation, or complications, surgery appears to offer distinct advantages for patients who do not achieve a CR after induction treatment.

This study is subject to several limitations. Firstly, it was a retrospective analysis, which inevitably introduces inherent biases. Secondly, the sample size was relatively small, which is attributable to the low incidence of CEC. Thirdly, due to the higher complication rates in patients with poor responses to chemoradiotherapy, salvage surgery was not pursued. Lastly, the follow-up duration was relatively short, and long-term survival data were not captured.

Our findings indicate that the “combined chemotherapy and immunotherapy screening” strategy, implemented as induction therapy before local treatment, effectively identifies suitable candidates for subsequent chemoradiotherapy or surgery. This approach not only enhances laryngeal preservation rates but also improves OS. Nevertheless, to validate its efficacy, further multicenter, prospective studies are essential for comparing this strategy with the current standard treatment of concurrent chemoradiotherapy.

## Appendix 2: OS and CSS of the entire patient cohort

.

## Appendix 3: OS and CSS of CR and non-CR patients

.

## Appendix 4: Comparison of survival between CR patients receiving chemoradiotherapy and non-CR patients receiving surgery

.

## Appendix 5: LFP survival rate for the entire patient cohort

.

## Appendix 6: LFP survival rates for CR and non-CR patients

.
