# Correction: Combined Chemotherapy and Immunotherapy Induction for Screening of Patients with Cervical Esophageal Carcinoma for Subsequent Local Treatment: A New Treatment Paradigm

**DOI:** 10.1245/s10434-024-16010-4

**Published:** 2024-08-02

**Authors:** Liang Dai, Ya-Ya Wu, Yan Sun, Rong Yu, Wan-Pu Yan, Yong-Bo Yang, Hong Cheng, Yi-Mei Gao, Bin Zhang, Ke-Neng Chen

**Affiliations:** 1 Key Laboratory of Carcinogenesis and Translational Research (Ministry of Education), The First Department of Thoracic Surgery, Peking University Cancer Hospital and Institute, Peking University School of Oncology, Beijing, China; 2 Department of Radiation Oncology, Peking University Cancer Hospital and Institute, Beijing, China; 3 Key Laboratory of Carcinogenesis and Translational Research (Ministry of Education), Department of Head and Neck Surgery, Peking University Cancer Hospital and Institute, Beijing, China

**Correction to: Annals of Surgical Oncology** 10.1245/s10434-024-15843-3

In the original online version of this article, Fig. 3 caption and Fig. 4 image were incorrect.

The original article was corrected.

